# Tandem electro-thermocatalytic system: redefining CO_2_ utilization efficiency in methane dry reforming

**DOI:** 10.1093/nsr/nwaf212

**Published:** 2025-05-22

**Authors:** Zhicheng Liu, Zaiku Xie

**Affiliations:** State Key Laboratory of Green Chemical Engineering and Industrial Catalysis, SINOPEC Shanghai Research Institute of Petrochemical Technology (SRIPT), China; State Key Laboratory of Green Chemical Engineering and Industrial Catalysis, SINOPEC Shanghai Research Institute of Petrochemical Technology (SRIPT), China; China Petrochemical Corporation (Sinopec Group), Beijing, China

Dry reforming of methane (DRM), a pivotal reaction for converting greenhouse gases CO_2_ and CH_4_ into syngas (CO and H_2_), has long been restricted by thermodynamic equilibrium limitations [1]. Traditional processes typically operate at a feed molar ratio of CO_2_ to CH_4_ of 1:1, yet many natural gas and biogas resources often contain >50% CO_2_ [2]. Consequently, substantial CO_2_ separation is required to access the necessary amount of CH_4_. From economic and scientific perspectives, it is essential to explore novel composite catalytic systems for high-carbon natural gas conversion without CO_2_ separation [3,4]. Specifically, can such systems overcome the intrinsic limitations in CO_2_ utilization efficiency observed in the conventional single-pathway DRM reaction?

The answer is yes! Recently, Wang and co-workers proposed a groundbreaking tandem electro-thermocatalytic system, integrating water electrolysis with thermal catalysis to overcome thermodynamic limitations, achieving super-dry reforming with unprecedented CO_2_ utilization efficiency and syngas selectivity [5].

**Figure 1.**
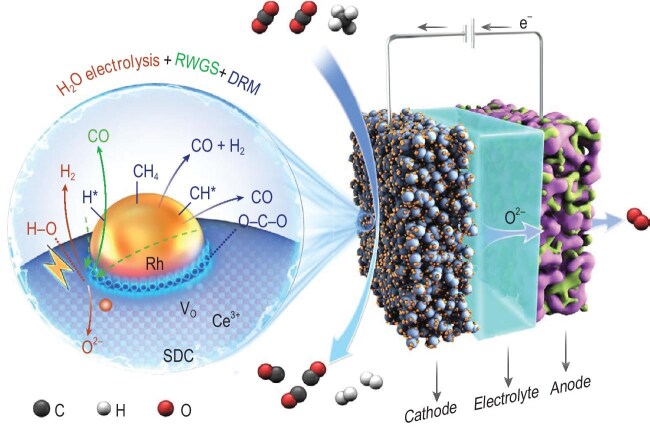
The tandem electro-thermocatalytic system. Schematic diagram of the tandem CH_4_ reforming process with the RWGS and H_2_O electrolysis reactions over the Ce^3+^–V_O_–Rh^δ+^ active sites at the cathode of the solid oxide electrolysis cell. SDC: Sm_x_Ce_1–x_O_2–δ_; V_O_: oxygen vacancy. Reproduced from Ref. [5] with permission.

The researchers ingeniously designed a three-step tandem process in an oxygen-ion-conducting electrolysis-membrane reactor (EMR) (Fig. 1). The tandem CH_4_ reforming process is integrated with the reverse water-gas shift (RWGS) reaction and water electrolysis, where water electrolysis shifts the equilibrium of the RWGS reaction, promoting syngas production and enhancing the apparent reducibility of CH_4_. This innovative design enables each CH_4_ molecule to consume up to as many as four CO_2_ molecules, significantly enhancing CO_2_ conversion efficiency. Experimental results demonstrate that at 800°C and a CO₂/CH₄ feed ratio of 4, the conversion rates of CO_2_ and CH_4_ reach 93.9% and 93.7%, respectively, while the selectivity for H_2_ exceeds 96% and that for CO approaches 100%.

Catalytic performance was further validated through 252-hour continuous operation, showcasing unwavering CO_2_ conversion and H_2_ selectivity. Advanced characterization techniques, including *in situ* STEM and NAP-XPS, revealed that the Rh/Sm_x_Ce_1–x_O_2–δ_ catalyst dynamically reconstructs Rh nanoparticles into (111) facets, exposing highly active sites for CH_4_ dissociation. Combined DFT calculations and operando spectroscopy elucidated a dual-pathway mechanism: (1) Thermal pathway: Ce^3+^-oxygen vacancy (V_O_)-Rh^δ+^ interfaces promote CO_2_ adsorption and dissociation. (2) Electrochemical pathway: H_2_ generation and O^2^⁻ removal at the cathode by transporting across the electrolyte membrane to the anode promotes the reduction of excess CO_2_, consequently shifting the equilibrium toward syngas production.

This work not only resolves the long-standing challenge of high-CO_2_ natural gas utilization but also holds transformative potential for carbon-neutral applications. By coupling electrolysis-driven renewable energy storage with syngas production, the technology promises sustainable routes for chemical manufacturing and hydrogen generation. The interface reconstruction-electrothermal synergy strategy established herein offers a paradigm for designing advanced catalysts in complex multi-field–coupled systems, heralding a new era for efficient carbon resource utilization.
